# 2-(Meth­oxy­imino)-2-{2-[(2-methyl­phenoxy)meth­yl]phen­yl}acetohydrazide

**DOI:** 10.1107/S160053681203070X

**Published:** 2012-07-10

**Authors:** Rajni Kant, Vivek K. Gupta, Kamini Kapoor, Chetan S. Shripanavar, Kaushik Banerjee

**Affiliations:** a X-ray Crystallography Laboratory, Post-Graduate Department of Physics and Electronics, University of Jammu, Jammu Tawi 180 006, India; b Department of Chemistry, Shivaji University, Kolhapur, 416 004, India; c National Research Centre for Grapes, Pune 412 307, India

## Abstract

In the title mol­ecule, C_17_H_19_N_3_O_3_, the dihedral angle between the two benzene rings is 57.17 (5)°. In the crystal, N—H⋯O and N—H⋯N hydrogen bonds connect mol­ecules to form chains along [001]. In addition, a weak C—H⋯π inter­action is observed.

## Related literature

For the biological activities of kresoxim-methyl {methyl 2(*E*)-meth­oxy­imino-2-[2-(2-tolyl­oxymeth­yl)phen­yl] acetate}, which is a starting material in the synthesis of the title compound, see: Anke *et al.* (1977 ▶); Balba (2007 ▶); Ichinari *et al.* (1999 ▶); Grossmann & Retzlaff (1997 ▶); Ypema (1998 ▶). For the crystal structure of kresoxim-methyl, see: Chopra *et al.* (2004 ▶).
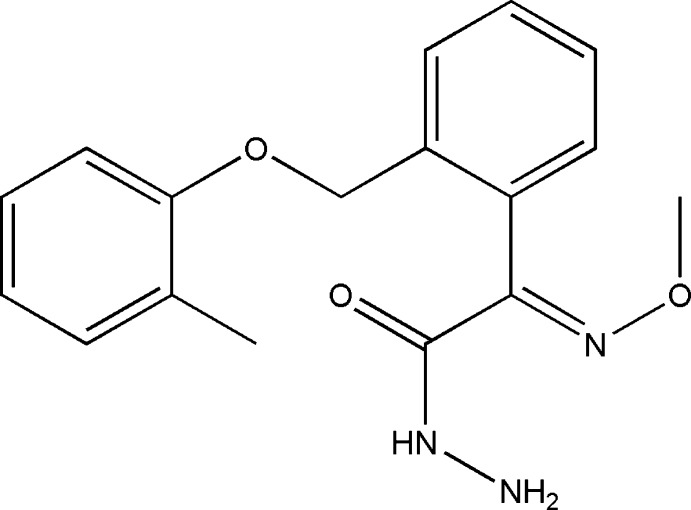


## Experimental

### 

#### Crystal data

C_17_H_19_N_3_O_3_
*M* *_r_* = 313.35
Monoclinic, 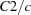

*a* = 21.4015 (6) Å
*b* = 20.7277 (4) Å
*c* = 7.6975 (2) Å
β = 109.103 (3)°
*V* = 3226.60 (14) Å^3^
*Z* = 8
Mo *K*α radiation
μ = 0.09 mm^−1^
*T* = 293 K
0.3 × 0.2 × 0.2 mm

#### Data collection

Oxford Diffraction Xcalibur Sapphire3 diffractometer
Absorption correction: multi-scan (*CrysAlis PRO*; Oxford Diffraction, 2010 ▶) *T*_min_ = 0.906, *T*_max_ = 1.000
55120 measured reflections
3174 independent reflections
2532 reflections with *I* > 2σ(*I*)
*R*_int_ = 0.042

#### Refinement

*R*[*F* ^2^ > 2σ(*F* ^2^)] = 0.040
*wR*(*F* ^2^) = 0.105
*S* = 1.02
3174 reflections
222 parameters
3 restraints
H atoms treated by a mixture of independent and constrained refinement
Δρ_max_ = 0.18 e Å^−3^
Δρ_min_ = −0.17 e Å^−3^

### 

Data collection: *CrysAlis PRO* (Oxford Diffraction, 2010 ▶); cell refinement: *CrysAlis PRO*; data reduction: *CrysAlis PRO*; program(s) used to solve structure: *SHELXS97* (Sheldrick, 2008 ▶); program(s) used to refine structure: *SHELXL97* (Sheldrick, 2008 ▶); molecular graphics: *ORTEP-3* (Farrugia, 1997 ▶); software used to prepare material for publication: *PLATON* (Spek, 2009 ▶).

## Supplementary Material

Crystal structure: contains datablock(s) I, global. DOI: 10.1107/S160053681203070X/lh5499sup1.cif

Structure factors: contains datablock(s) I. DOI: 10.1107/S160053681203070X/lh5499Isup2.hkl

Supplementary material file. DOI: 10.1107/S160053681203070X/lh5499Isup3.cml

Additional supplementary materials:  crystallographic information; 3D view; checkCIF report

## Figures and Tables

**Table 1:** Hydrogen-bond geometry (Å, °) *Cg* refers to the centroid of the C8–C13 phenyl ring.

*D*—H⋯*A*	*D*—H	H⋯*A*	*D*⋯*A*	*D*—H⋯*A*
N7—H72⋯O1^i^	0.86 (2)	2.40 (2)	3.220 (2)	158 (2)
N6—H61⋯N7^ii^	0.87 (2)	2.34 (1)	3.191 (2)	166 (1)
C5—H5*B*⋯*Cg* ^iii^	0.96	2.69	3.461 (2)	138

Symmetry codes: (i) 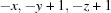; (ii) 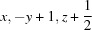; (iii) 
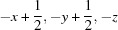
.

**Fig. 1.**
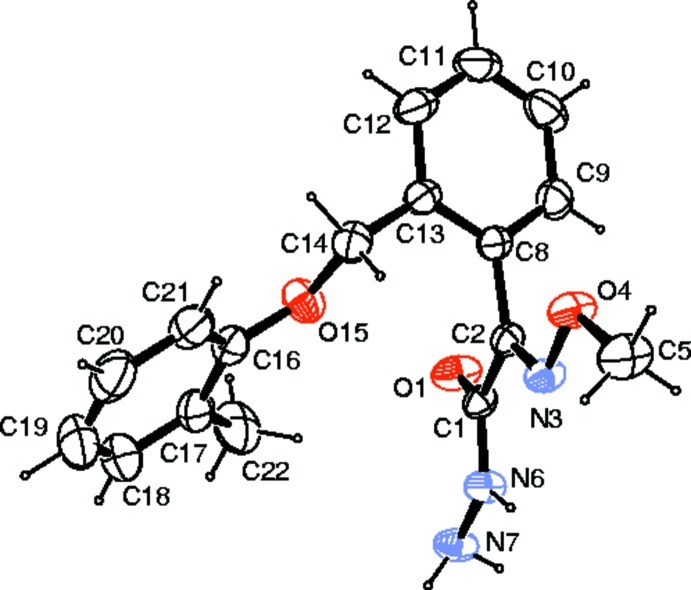
The molecular structure of (I) with ellipsoids drawn at the 40% probability level. H atoms are shown as small spheres of arbitrary radii.

**Fig. 2.**
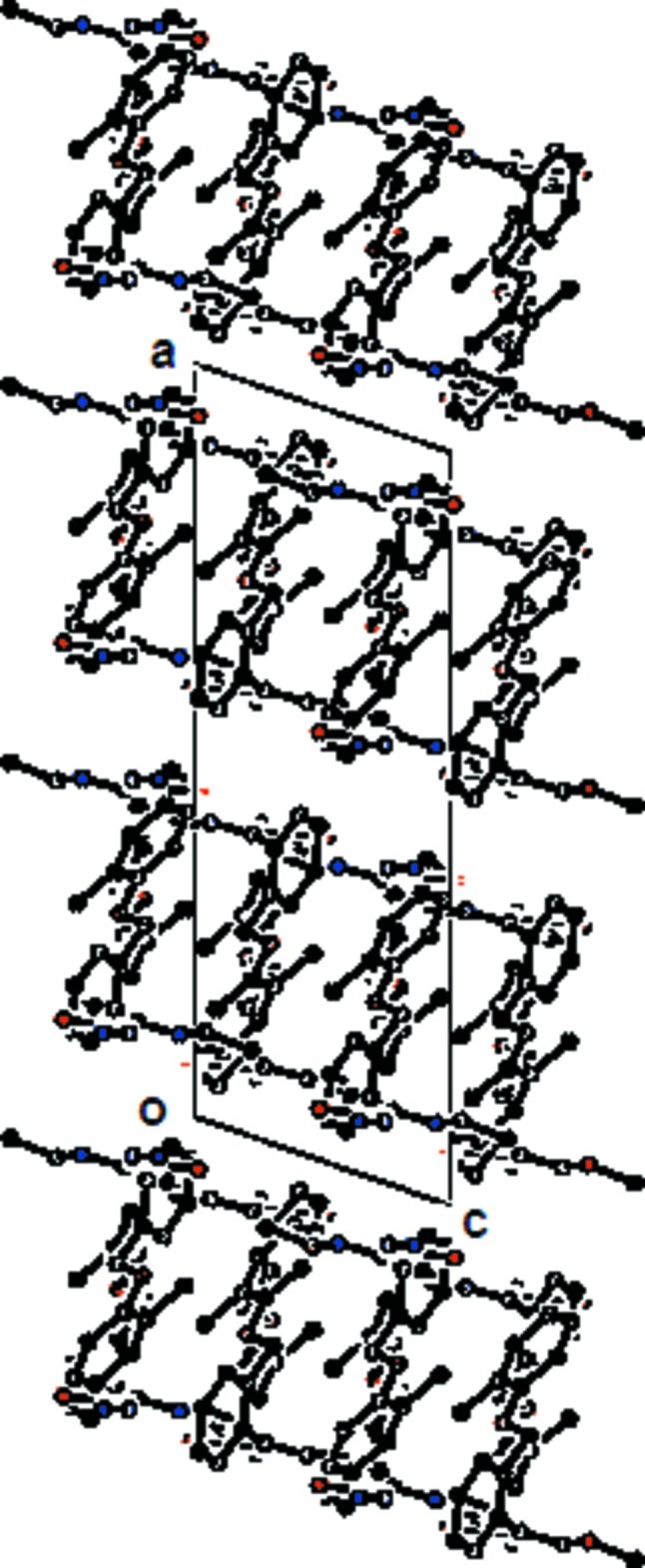
Part of the crystal structure viewed along the b axis.

## supplementary crystallographic information

### Comment

Kresoxim-methyl is an active agrochemical (Chopra *et al.*, 2004), strobilurin fungicide (Anke *et al.*, 1977), with broad spectrum biological activity (Ypema, 1998; Ichinari *et al.*, 1999; Grossmann & Retzlaff 1997). This type of compound is easily metabolized in nature as well as in living systems, and that is the reason studies on their fate in soil, plants and animal systems (Balba, 2007) are very important. Herein, we present the crystal structure of the title compound (I) which was synthesized from kresoxim-methyl.

In (I) (Fig. 1), all bond lengths and angles are normal and correspond to those observed in the related structure (Chopra *et al.*, 2004). The dihedral angles formed by the two benzene rings (C8-C13/C16-C21) is 57.17 (5)°. In the crystal, molecules are connected by N—H···O and N—H···N hydrogen bonds into chains along [001] (Fig. 2). In addition a weak C—H···π interaction is observed.

### Experimental

Kresoxim-methyl (0.313 g, 0.001 mol) was dissolved in 5 ml methanol and to it hydrazine hydrate (0.1 g, 0.002 mol) solution was added and refluxed at 343K for 1 h. The reaction mixture was then cooled and solvent was removed under reduced presser to give a solid product. The compound was dissolved in methanol, and by the process of slow evaporation this crystalline compound was separated out, m.p. 397K.

### Refinement

H atoms bonded to N atoms were located in a difference map and refined independently with the constraint of N—H = 0.86 (1)Å. Other H atoms were positioned geometrically and were treated as riding on their parent C atoms, with C—H distances of 0.93–0.97 Å and with *U*_iso_(H) = 1.2*U*_eq_(C) or 1.5*U*_eq_(methyl C).

### Crystal data

**Table:**

C_17_H_19_N_3_O_3_	*F*(000) = 1328
*M**_r_* = 313.35	*D*_x_ = 1.290 Mg m^−^^3^
Monoclinic, *C*2/*c*	Mo *K*α radiation, λ = 0.71073 Å
Hall symbol: -C 2yc	Cell parameters from 21236 reflections
*a* = 21.4015 (6) Å	θ = 3.5–29.1°
*b* = 20.7277 (4) Å	µ = 0.09 mm^−^^1^
*c* = 7.6975 (2) Å	*T* = 293 K
β = 109.103 (3)°	Block, white
*V* = 3226.60 (14) Å^3^	0.3 × 0.2 × 0.2 mm
*Z* = 8	

### Data collection

**Table:**

Oxford Diffraction Xcalibur Sapphire3 diffractometer	3174 independent reflections
Radiation source: fine-focus sealed tube	2532 reflections with *I* > 2σ(*I*)
Graphite monochromator	*R*_int_ = 0.042
Detector resolution: 16.1049 pixels mm^-1^	θ_max_ = 26.0°, θ_min_ = 3.5°
ω scan	*h* = −26→26
Absorption correction: multi-scan (*CrysAlis PRO*; Oxford Diffraction, 2010)	*k* = −25→25
*T*_min_ = 0.906, *T*_max_ = 1.000	*l* = −9→9
55120 measured reflections	

### Refinement

**Table:**

Refinement on *F*^2^	Primary atom site location: structure-invariant direct methods
Least-squares matrix: full	Secondary atom site location: difference Fourier map
*R*[*F*^2^ > 2σ(*F*^2^)] = 0.040	Hydrogen site location: inferred from neighbouring sites
*wR*(*F*^2^) = 0.105	H atoms treated by a mixture of independent and constrained refinement
*S* = 1.02	*w* = 1/[σ^2^(*F*_o_^2^) + (0.0448*P*)^2^ + 1.9045*P*] where *P* = (*F*_o_^2^ + 2*F*_c_^2^)/3
3174 reflections	(Δ/σ)_max_ = 0.001
222 parameters	Δρ_max_ = 0.18 e Å^−^^3^
3 restraints	Δρ_min_ = −0.17 e Å^−^^3^

### Special details

**Table:**

Experimental. *CrysAlis PRO*, Oxford Diffraction Ltd., Version 1.171.34.40 (release 27–08-2010 CrysAlis171. NET) (compiled Aug 27 2010,11:50:40) Empirical absorption correction using spherical harmonics, implemented in SCALE3 ABSPACK scaling algorithm.
Geometry. All e.s.d.'s (except the e.s.d. in the dihedral angle between two l.s. planes) are estimated using the full covariance matrix. The cell e.s.d.'s are taken into account individually in the estimation of e.s.d.'s in distances, angles and torsion angles; correlations between e.s.d.'s in cell parameters are only used when they are defined by crystal symmetry. An approximate (isotropic) treatment of cell e.s.d.'s is used for estimating e.s.d.'s involving l.s. planes.
Refinement. Refinement of *F*^2^ against ALL reflections. The weighted *R*-factor *wR* and goodness of fit *S* are based on *F*^2^, conventional *R*-factors *R* are based on *F*, with *F* set to zero for negative *F*^2^. The threshold expression of *F*^2^ > σ(*F*^2^) is used only for calculating *R*-factors(gt) *etc*. and is not relevant to the choice of reflections for refinement. *R*-factors based on *F*^2^ are statistically about twice as large as those based on *F*, and *R*- factors based on ALL data will be even larger.

### Fractional atomic coordinates and isotropic or equivalent isotropic displacement parameters (Å^2^)

**Table:**

	*x*	*y*	*z*	*U*_iso_*/*U*_eq_	
N3	0.10242 (6)	0.35418 (5)	0.94034 (15)	0.0383 (3)	
N6	0.08078 (6)	0.46145 (6)	0.74149 (16)	0.0391 (3)	
N7	0.06911 (8)	0.51996 (6)	0.64119 (18)	0.0453 (3)	
O15	0.23561 (6)	0.35236 (5)	0.70574 (15)	0.0510 (3)	
O4	0.11743 (6)	0.29666 (5)	1.03992 (13)	0.0466 (3)	
O1	0.06825 (6)	0.40146 (5)	0.48718 (13)	0.0521 (3)	
C17	0.28955 (8)	0.44911 (8)	0.6901 (2)	0.0519 (4)	
C18	0.34451 (11)	0.48795 (10)	0.7505 (3)	0.0694 (6)	
H18	0.3437	0.5282	0.6965	0.083*	
C19	0.40054 (11)	0.46909 (12)	0.8882 (3)	0.0808 (7)	
H19	0.4372	0.4961	0.9252	0.097*	
C20	0.40213 (10)	0.41013 (11)	0.9710 (3)	0.0706 (6)	
H20	0.4400	0.3972	1.0645	0.085*	
C21	0.34756 (8)	0.36974 (9)	0.9158 (2)	0.0546 (4)	
H21	0.3484	0.3300	0.9728	0.066*	
C16	0.29186 (8)	0.38918 (8)	0.7750 (2)	0.0444 (4)	
C22	0.22894 (10)	0.47007 (10)	0.5382 (3)	0.0728 (6)	
H22A	0.2339	0.5142	0.5069	0.109*	
H22B	0.1910	0.4662	0.5780	0.109*	
H22C	0.2231	0.4432	0.4324	0.109*	
C14	0.23124 (8)	0.29575 (7)	0.8055 (2)	0.0453 (4)	
H14A	0.2306	0.3074	0.9270	0.054*	
H14B	0.2695	0.2685	0.8198	0.054*	
C13	0.16963 (7)	0.26008 (7)	0.70408 (19)	0.0382 (3)	
C12	0.17407 (9)	0.20035 (8)	0.6249 (2)	0.0523 (4)	
H12	0.2155	0.1838	0.6353	0.063*	
C11	0.11869 (11)	0.16554 (8)	0.5321 (3)	0.0603 (5)	
H11	0.1228	0.1257	0.4814	0.072*	
C10	0.05738 (10)	0.18966 (9)	0.5143 (2)	0.0593 (5)	
H10	0.0198	0.1663	0.4507	0.071*	
C9	0.05121 (8)	0.24886 (8)	0.5911 (2)	0.0473 (4)	
H9	0.0094	0.2650	0.5783	0.057*	
C8	0.10689 (7)	0.28413 (6)	0.68670 (18)	0.0347 (3)	
C2	0.09848 (7)	0.34665 (6)	0.77269 (18)	0.0320 (3)	
C5	0.11414 (11)	0.30815 (9)	1.2204 (2)	0.0608 (5)	
H5A	0.1481	0.3382	1.2840	0.091*	
H5B	0.1205	0.2683	1.2873	0.091*	
H5C	0.0716	0.3257	1.2106	0.091*	
C1	0.08095 (7)	0.40622 (7)	0.65305 (18)	0.0334 (3)	
H61	0.0840 (8)	0.4629 (7)	0.8568 (13)	0.041 (4)*	
H71	0.0964 (9)	0.5477 (8)	0.707 (3)	0.081 (7)*	
H72	0.0295 (6)	0.5333 (11)	0.627 (3)	0.090 (8)*	

### Atomic displacement parameters (Å^2^)

**Table:**

	*U*^11^	*U*^22^	*U*^33^	*U*^12^	*U*^13^	*U*^23^
N3	0.0523 (7)	0.0327 (6)	0.0321 (6)	0.0099 (5)	0.0169 (5)	0.0061 (5)
N6	0.0587 (8)	0.0320 (6)	0.0273 (6)	0.0039 (5)	0.0149 (6)	0.0027 (5)
N7	0.0656 (10)	0.0306 (7)	0.0381 (7)	0.0039 (7)	0.0147 (7)	0.0031 (5)
O15	0.0515 (7)	0.0461 (6)	0.0510 (7)	−0.0027 (5)	0.0109 (5)	0.0143 (5)
O4	0.0752 (8)	0.0367 (6)	0.0327 (5)	0.0146 (5)	0.0244 (5)	0.0094 (4)
O1	0.0866 (9)	0.0427 (6)	0.0291 (5)	0.0151 (6)	0.0215 (5)	0.0035 (4)
C17	0.0549 (10)	0.0522 (10)	0.0582 (10)	−0.0013 (8)	0.0317 (8)	0.0044 (8)
C18	0.0698 (14)	0.0621 (12)	0.0891 (15)	−0.0130 (10)	0.0435 (12)	−0.0013 (11)
C19	0.0598 (13)	0.0881 (16)	0.1030 (18)	−0.0239 (12)	0.0382 (13)	−0.0213 (14)
C20	0.0448 (11)	0.0892 (16)	0.0760 (13)	0.0057 (10)	0.0172 (9)	−0.0183 (12)
C21	0.0504 (10)	0.0566 (10)	0.0577 (10)	0.0075 (8)	0.0188 (8)	−0.0050 (8)
C16	0.0443 (9)	0.0451 (9)	0.0491 (9)	0.0019 (7)	0.0226 (7)	−0.0006 (7)
C22	0.0763 (14)	0.0638 (12)	0.0795 (14)	−0.0005 (10)	0.0271 (11)	0.0313 (10)
C14	0.0445 (9)	0.0439 (9)	0.0489 (9)	0.0101 (7)	0.0171 (7)	0.0141 (7)
C13	0.0481 (9)	0.0341 (7)	0.0373 (7)	0.0075 (6)	0.0209 (6)	0.0088 (6)
C12	0.0712 (12)	0.0402 (9)	0.0566 (10)	0.0157 (8)	0.0360 (9)	0.0073 (7)
C11	0.0990 (16)	0.0358 (9)	0.0598 (11)	−0.0025 (9)	0.0446 (11)	−0.0069 (8)
C10	0.0804 (13)	0.0514 (10)	0.0523 (10)	−0.0246 (9)	0.0303 (9)	−0.0129 (8)
C9	0.0503 (9)	0.0516 (9)	0.0451 (9)	−0.0055 (7)	0.0225 (7)	−0.0034 (7)
C8	0.0457 (8)	0.0328 (7)	0.0299 (7)	0.0028 (6)	0.0184 (6)	0.0041 (5)
C2	0.0339 (7)	0.0331 (7)	0.0307 (7)	0.0043 (5)	0.0130 (6)	0.0027 (5)
C5	0.0999 (15)	0.0554 (10)	0.0335 (8)	0.0123 (10)	0.0307 (9)	0.0089 (7)
C1	0.0367 (7)	0.0359 (7)	0.0297 (7)	0.0058 (6)	0.0136 (6)	0.0028 (5)

### Geometric parameters (Å, º)

**Table:**

N3—C2	1.2750 (17)	C22—H22A	0.9600
N3—O4	1.3968 (14)	C22—H22B	0.9600
N6—C1	1.3326 (17)	C22—H22C	0.9600
N6—N7	1.4153 (17)	C14—C13	1.490 (2)
N6—H61	0.868 (9)	C14—H14A	0.9700
N7—H71	0.858 (10)	C14—H14B	0.9700
N7—H72	0.864 (10)	C13—C12	1.397 (2)
O15—C16	1.3767 (19)	C13—C8	1.397 (2)
O15—C14	1.4222 (17)	C12—C11	1.372 (3)
O4—C5	1.4335 (18)	C12—H12	0.9300
O1—C1	1.2192 (16)	C11—C10	1.369 (3)
C17—C18	1.375 (3)	C11—H11	0.9300
C17—C16	1.397 (2)	C10—C9	1.387 (2)
C17—C22	1.498 (3)	C10—H10	0.9300
C18—C19	1.372 (3)	C9—C8	1.387 (2)
C18—H18	0.9300	C9—H9	0.9300
C19—C20	1.374 (3)	C8—C2	1.4926 (18)
C19—H19	0.9300	C2—C1	1.5123 (18)
C20—C21	1.386 (3)	C5—H5A	0.9600
C20—H20	0.9300	C5—H5B	0.9600
C21—C16	1.383 (2)	C5—H5C	0.9600
C21—H21	0.9300		
			
C2—N3—O4	112.21 (11)	C13—C14—H14A	109.8
C1—N6—N7	119.14 (11)	O15—C14—H14B	109.8
C1—N6—H61	122.7 (10)	C13—C14—H14B	109.8
N7—N6—H61	117.9 (10)	H14A—C14—H14B	108.3
N6—N7—H71	106.7 (15)	C12—C13—C8	118.36 (15)
N6—N7—H72	109.5 (16)	C12—C13—C14	119.56 (14)
H71—N7—H72	108 (2)	C8—C13—C14	122.07 (13)
C16—O15—C14	116.85 (12)	C11—C12—C13	121.52 (16)
N3—O4—C5	108.33 (11)	C11—C12—H12	119.2
C18—C17—C16	117.83 (17)	C13—C12—H12	119.2
C18—C17—C22	121.37 (17)	C10—C11—C12	119.78 (15)
C16—C17—C22	120.80 (16)	C10—C11—H11	120.1
C19—C18—C17	122.0 (2)	C12—C11—H11	120.1
C19—C18—H18	119.0	C11—C10—C9	120.17 (17)
C17—C18—H18	119.0	C11—C10—H10	119.9
C18—C19—C20	119.6 (2)	C9—C10—H10	119.9
C18—C19—H19	120.2	C10—C9—C8	120.50 (16)
C20—C19—H19	120.2	C10—C9—H9	119.7
C19—C20—C21	120.3 (2)	C8—C9—H9	119.7
C19—C20—H20	119.8	C9—C8—C13	119.66 (13)
C21—C20—H20	119.8	C9—C8—C2	119.06 (13)
C16—C21—C20	119.22 (18)	C13—C8—C2	121.27 (13)
C16—C21—H21	120.4	N3—C2—C8	125.28 (12)
C20—C21—H21	120.4	N3—C2—C1	115.94 (11)
O15—C16—C21	124.22 (15)	C8—C2—C1	118.73 (11)
O15—C16—C17	114.76 (14)	O4—C5—H5A	109.5
C21—C16—C17	121.01 (16)	O4—C5—H5B	109.5
C17—C22—H22A	109.5	H5A—C5—H5B	109.5
C17—C22—H22B	109.5	O4—C5—H5C	109.5
H22A—C22—H22B	109.5	H5A—C5—H5C	109.5
C17—C22—H22C	109.5	H5B—C5—H5C	109.5
H22A—C22—H22C	109.5	O1—C1—N6	124.57 (13)
H22B—C22—H22C	109.5	O1—C1—C2	119.84 (12)
O15—C14—C13	109.25 (12)	N6—C1—C2	115.59 (11)
O15—C14—H14A	109.8		
			
C2—N3—O4—C5	−174.13 (14)	C12—C11—C10—C9	−0.5 (3)
C16—C17—C18—C19	0.7 (3)	C11—C10—C9—C8	−0.1 (2)
C22—C17—C18—C19	−179.34 (19)	C10—C9—C8—C13	0.7 (2)
C17—C18—C19—C20	−0.9 (3)	C10—C9—C8—C2	−178.03 (13)
C18—C19—C20—C21	0.1 (3)	C12—C13—C8—C9	−0.6 (2)
C19—C20—C21—C16	0.8 (3)	C14—C13—C8—C9	−179.88 (13)
C14—O15—C16—C21	10.5 (2)	C12—C13—C8—C2	178.12 (12)
C14—O15—C16—C17	−170.36 (13)	C14—C13—C8—C2	−1.2 (2)
C20—C21—C16—O15	178.15 (15)	O4—N3—C2—C8	1.5 (2)
C20—C21—C16—C17	−1.0 (2)	O4—N3—C2—C1	178.76 (11)
C18—C17—C16—O15	−178.97 (15)	C9—C8—C2—N3	96.60 (18)
C22—C17—C16—O15	1.1 (2)	C13—C8—C2—N3	−82.08 (18)
C18—C17—C16—C21	0.2 (2)	C9—C8—C2—C1	−80.61 (16)
C22—C17—C16—C21	−179.71 (17)	C13—C8—C2—C1	100.71 (15)
C16—O15—C14—C13	−176.27 (12)	N7—N6—C1—O1	−4.0 (2)
O15—C14—C13—C12	111.95 (15)	N7—N6—C1—C2	175.93 (13)
O15—C14—C13—C8	−68.73 (17)	N3—C2—C1—O1	−170.98 (14)
C8—C13—C12—C11	−0.1 (2)	C8—C2—C1—O1	6.5 (2)
C14—C13—C12—C11	179.25 (14)	N3—C2—C1—N6	9.09 (19)
C13—C12—C11—C10	0.6 (3)	C8—C2—C1—N6	−173.44 (13)

### Hydrogen-bond geometry (Å, º)

Please define Cg

**Table:**

*D*—H···*A*	*D*—H	H···*A*	*D*···*A*	*D*—H···*A*
N7—H72···O1^i^	0.86 (2)	2.40 (2)	3.220 (2)	158 (2)
N6—H61···N7^ii^	0.87 (2)	2.34 (1)	3.191 (2)	166 (1)
C5—H5*B*···*Cg*^iii^	0.96	2.69	3.461 (2)	138

Symmetry codes: (i) −*x*, −*y*+1, −*z*+1; (ii) *x*, −*y*+1, *z*+1/2; (iii) −*x*+1/2, −*y*+1/2, −*z*.
